# Special Issue “Extragastric Disorders of *Helicobacter pylori* Infection: From Diagnosis to Treatment”: Editorial

**DOI:** 10.3390/microorganisms11030677

**Published:** 2023-03-07

**Authors:** Michael Doulberis, Apostolis Papaefthymiou, Jannis Kountouras

**Affiliations:** 1 Gastroklinik, Private Gastroenterological Practice, 8810 Horgen, Switzerland; 2 Gastroenterology Section, Hirslanden Klinik im Park, 8027 Zurich, Switzerland; 3 Division of Gastroenterology and Hepatology, Medical University Department, Kantonsspital Aarau, 5001 Aarau, Switzerland; 4 Department of Internal Medicine, Second Medical Clinic, Ippokration Hospital, Aristotle University of Thessaloniki, 54642 Thessaloniki, Greece; 5 First Laboratory of Pharmacology, School of Medicine, Aristotle University of Thessaloniki, 54124 Thessaloniki, Greece; 6 Pancreaticobiliary Medicine Unit, University College London Hospitals (UCLH), London W1W 6DN, UK

Gut microorganisms represent a very attractive field of contemporary biomedical research since they exhibit complex interactions with their host and shape immunity in health and disease [1,2]. Among their other functions, they also orchestrate and regulate a plethora of metabolic, endocrine, and neuronal pathways [3].

In this respect, *Helicobacter pylori (H. pylori)* belongs to the non-commensal gut microbiome, and beyond its very well substantiated pathogenicity in the stomach in terms of peptic ulceration and gastric adenocarcinoma/MALT, it possess multiple extragastric manifestations with varying degrees of available evidence [3,4,5,6]. Moreover, *H. pylori*-related extragastric upper and lower gastrointestinal tract disorders include, for instance, oesophageal and colorectal neoplasms in certain populations [7,8,9]. Further systemic features of *H. pylori* infection include, for example, metabolic syndrome (MetS)-associated disorders, namely type 2 diabetes mellitus [10], arterial hypertension (AH) [11], and/or non-alcoholic fatty liver disease, recently renamed as metabolic (dysfunction)-associated fatty liver disease [12,13]. Moreover, among others, neurodegenerative, cardiovascular, ophthalmic, kidney, respiratory, and allergic diseases [4,14,15,16] have been also implicated to *H. pylori’s* possible systematic pathogenicity, albeit the actual burden and relationship remain, as of yet, unknown. Not only *H. pylori* but also other *Helicobacter* species have been investigated in basic research studies, and it has been shown that they can distantly influence the immune system and anatomically remote tissues and organs [15,17]. *H. pylori*-activated innate and adaptive immune systems contribute to *H. pylori*-related distant systemic pathologies [18].

Within this Special Issue entitled “Extragastric Disorders of *Helicobacter pylori* Infection: From Diagnosis to Treatment”, our primary aim is to provide a further contribution to insights into the relationship between *H. pylori* infection and systemic pathologies. Regarding the intended impact of this Special Issue, motivating further relevant studies would feasibly constitute, in the future, more visibility and acceptance of this pleotropic pathogenicity of *H. pylori* as a risk factor and common denominator in many extragastric pathologies. This Special Issue includes the following four articles [11,19,20,21], two original articles and two reviews:

Durazzo et al. [20] performed a systematic review regarding the potential association between *H. pylori* and respiratory diseases. Out of 227 initially yielded results, in conclusion, 30 studies were eligible for further evaluation based on the set criteria. The results were divided into certain respiratory diseases, including asthma, chronic obstructive pulmonary disease, bronchiectasis, lung cancer, tuberculosis, cystic fibrosis, and sarcoidosis. The authors deduced that the vast majority of studies were retrospective case–control studies with a small number of participants and great heterogeneity in their study design, e.g., the method used for the diagnosis of *H. pylori* infection. Therefore, the question as to what extent *H. pylori* is a risk factor or a protective factor could not be answered. Three pathogenetic hypotheses were suggested, i.e., autoantibodies induced by *H. pylori* components (molecular mimicry), host immune response (cytokine cascade), and acid gastric content aspiration or inhalation (direct damage).

Focusing on the current, crucial topic of the failure to eradicate *H. pylori* [22], Ozeki et al. [19] investigated the relationship between the difficulty to eradicate *H. pylori* and drinking habits and allergies. In view of previous evidence supporting bacterial eradication failure with high immunoglobulin E (IgE) levels, the authors recruited 250 individuals and collected questionnaires along with IgE blood samples. The results revealed that participants with allergic diseases and those with high alcohol intake were characterised by statistically significantly higher IgE levels. High IgE levels were therefore regarded as a risk factor for the failure to eradicate *H. pylori* in connection with drinking habits and alcohol consumption.

We performed a retrospective study [21] in bariatric subjects with the aim of assessing the impact of *H. pylori* on metabolic parameters and premalignant gastric mucosa histological lesions. After reviewing 94,304 patient cases, 116 eligible patients who had undergone bariatric surgery with their *H. pylori* status also available satisfied the inclusion criteria. In the relevant results, the following factors were included: the presence of gastric mucosa atrophy and intestinal metaplasia was statistically significant for the *H. pylori* group (*p* = 0.006 and *p* < 0.0001, respectively). Moreover, *H. pylori*-positive patients had statistically higher AH (*p* = 0.033). The same applied to the so-called homeostatic model for the assessment of insulin resistance (*p* < 0.001). Lastly, in a multivariate analysis, including AH, gastric mucosa atrophy, and intestinal metaplasia as variables, statistical significance remained only for intestinal metaplasia (*p* = 0.001).

Moreover, we performed a narrative review to reveal the possible impact of *H. pylori* on AH, as a component of MetS. Three pathogenetic pillars were suggested: metabolic pathways (implicating insulin resistance, dyslipidemia, inositol, monocytes, and fibroblasts), dietary factors (implicating salt consumption), and inflammatory factors (implicating inflammatory mediators—cytokines such as tumour necrosis α, interferon γ, and interleukins 1, 6, and 8). Conclusively, although an etiological linkage cannot (yet?) be proven, an emerging body of evidence emanating from preclinical and clinical studies supports an association between *H. pylori* infection and AH.

As demonstrated herein, *H. pylori* as gut microbiota processes several hallmarks, including carcinogenesis which may affect distant organs and shape the immune system in several settings. However, given the lack of mechanistical studies and large-scale clinical studies to support etiological involvement, we may hopefully inspire future researchers to shed light on the real burden, as insights into the association will have important clinical consequences.

## Data Availability

Not applicable.
